# Neural mapping of prepulse‐induced startle reflex modulation as indices of sensory information processing in healthy and clinical populations: A systematic review

**DOI:** 10.1002/hbm.25631

**Published:** 2021-08-20

**Authors:** Laura F. Naysmith, Veena Kumari, Steven C. R. Williams

**Affiliations:** ^1^ Centre for Neuroimaging Sciences, Institute of Psychiatry, Psychology and Neuroscience King's College London London UK; ^2^ Department of Psychology, Institute of Psychiatry, Psychology and Neuroscience King's College London London UK; ^3^ Centre for Cognitive Neuroscience, College of Health Medicine and Life Sciences, Brunel University London UK

**Keywords:** functional magnetic resonance imaging, prepulse facilitation, prepulse inhibition, sensory information processing, startle reflex

## Abstract

Startle reflex is modulated when a weaker sensory stimulus (“prepulse”) precedes a startling stimulus (“pulse”). Prepulse Inhibition (PPI) is the attenuation of the startle reflex (prepulse precedes pulse by 30–500 ms), whereas Prepulse Facilitation (PPF) is the enhancement of the startle reflex (prepulse precedes pulse by 500–6000 ms). Here, we critically appraise human studies using functional neuroimaging to establish brain regions associated with PPI and PPF. Of 10 studies, nine studies revealed thalamic, striatal and frontal lobe activation during PPI in healthy groups, and activation deficits in the cortico‐striato‐pallido‐thalamic circuitry in schizophrenia (three studies) and Tourette Syndrome (two studies). One study revealed a shared network for PPI and PPF in frontal regions and cerebellum, with PPF networks recruiting superior medial gyrus and cingulate cortex. The main gaps in the literature are (i) limited PPF research and whether PPI and PPF operate on separate/shared networks, (ii) no data on sex differences in neural underpinnings of PPI and PPF, and (iii) no data on neural underpinnings of PPI and PPF in other clinical disorders.

## 1 INTRODUCTION

The startle reflex is a rapid and involuntary motor response which is elicited by a sudden and intense sensory stimulus, such as a loud noise or unexpected touch to the body (Graham, 1975). As a protection/defensive response (Yeomans, Li, Scott, & Frankland, 2002), the startle response has been shown to become more intense when presented with threat of pain (Bradley, Silakowski, & Lang, 2008; Davis, 1989) and fear (Davis, Falls, Campeau, & Kim, 1993; Grillon et al., 1999). The startle reflex is seen across all mammals with a consistent motor response (Koch, 1999), typically as an eyeblink response in humans, and as a whole‐body motor response in animals (Geyer, Swerdlow, Mansbach, & Braff, 1990; Swerdlow, Braff, & Geyer, 1999).

Although an automatic reflex, the startle response can be modified. For example, startle response will decrease with habituation (Hoffman & Searle, 1968; Tighe & Leaton, 2016) and increase with fear (Davis, Hitchcock, & Rosen, 1988) and negative affect (Bradley, Lang, & Cuthbert, 1993). Prepulse‐pulse pairing, whereby a weak sensory stimulus (“prepulse”) precedes a startling sensory stimulus (“pulse”), also modulates the startle reflex (Graham, 1975), demonstrating flexibility and plasticity of this brainstem reflex. Prepulse‐pulse pairing can inhibit, facilitate, or have no effect on the startle response, depending on the length of time between the pulse and prepulse (the stimulus onset asynchrony, SoA). The estimated temporal window for inhibiting the startle reflex, known as Prepulse Inhibition (PPI), is 30–500 ms (Graham, 1975). In humans, amplitude of the eye blink has been demonstrated to be inhibited by 50% or more (Hazlett et al., 2008), with maximal inhibition at 120 ms (Hazlett et al., 2001). To facilitate the startle reflex, known as Prepulse Facilitation (PPF), the SoA should be longer, at >500–6000 ms in humans (see Putnam & Vanman, 1999).

### 1.1 Prepulse inhibition (PPI)

PPI is believed to be an operational measure of sensorimotor gating (Braff & Geyer, 1990) that inhibits processing of incoming stimulus (the pulse) at the same time as processing of an earlier presented stimulus (the prepulse) is ongoing, and thus protects processing of the prepulse (Graham, 1975; Graham & Murray, 1977). There is empirical support for the theory that PPI reflects protection of initial processing of the prepulse (e.g., Kumari, Gray, Gupta, Luscher, & Sharma, 2003; Norris & Blumenthal, 1996; Postma, Kumari, Hines, & Gray, 2001), though this may not be the only or complete explanation (Blumenthal, Reynolds, & Spence, 2015). PPI has demonstrated cross‐modal and cross‐species reliability (Swerdlow et al., 1999), high face, predictive and construct validity (Panther et al., 2019), and has provided insight into preattentive mechanisms of sensory information processing (Hazlett et al., 1998, 2008). Sensorimotor gating impairments appear to be key characteristics to a number of clinical disorders, therefore PPI has been used to explore sensory processing and information processing disturbances, such as in schizophrenia (Geyer et al., 1990; Kumari, Gray, Geyer, et al., 2003; Kumari et al., 2007), Tourette's Syndrome (TS; Buse, Beste, Herrmann, & Roessner, 2016; Swerdlow, Geyer, & Braff, 2001; Zebardast et al., 2013), Obsessive Compulsive Disorder (OCD) (Ahmari, Risbrough, Geyer, & Simpson, 2012; Swerdlow, Benbow, Zisook, Geyer, & Braff, 1993), Bipolar disorder (Perry, Minassian, Feifel, & Braff, 2001), Enuresis in children (Ornitz, Hanna, & de Traversay, 1992; Baeyens, Roeyers, Van Erdeghem, Hoebeke, & Vande Walle, 2007), Alzheimer's disease (Hejl, Glenthøj, Mackeprang, Hemmingsen, & Waldemar, 2004) and Huntington' disease (Swerdlow et al., 1995).

Early animal research has identified a cortical–subcortical pathway from the brainstem which mediates PPI, known as the cortico‐striato‐pallido‐thalamic (CSPT) circuit (Swerdlow & Geyer, 1998). The functional neuroanatomy of the startle reflex include: the cochlear nuclei, caudal pontine ventricular nucleus, inferior colliculus (for auditory stimulation) and superior colliculus (for visual and tactile stimulation), pedunculopontine and laterodorsal tegmental nuclei (Fendt, Li, & Yeomans, 2001). Rodent models have mapped CSPT circuitry (Swerdlow, Geyer, & Braff, 2001), highlighting the roles of the frontal cortex, thalamus, hippocampus, basolateral amygdala, ventral pallidum and nucleus accumbens. This suggests modulatory activity of the automatic brainstem reflex via a top‐down process. Several neurotransmitters, including dopamine, gamma aminobutyric acid (GABA), noradrenaline, serotonin, acetylcholine and glutamate, are thought to mediate PPI (Geyer, Krebs‐Thomson, Braff, & Swerdlow, 2001; Koch, 1999; Swerdlow, Braff, & Geyer, 2016). A number of studies have highlighted significant differences in PPI with dopaminergic, glutaminergic and serotonergic dysfunction, and this has been linked to neuropsychiatric disorders, such as schizophrenia (see Swerdlow, Caine, Braff, & Geyer, 1992).

Over the past two decades, technological advances in the field of Neuroscience have accelerated research with neuroimaging techniques to map the structural and functional underpinnings of PPI in humans. This has been particularly important for identifying normal functioning neural activity in healthy human participants, in comparison to clinical populations which show impairments in PPI, such as schizophrenia (Hazlett et al., 2008; Kumari, Gray, Geyer, et al., 2003).

### 1.2 Prepulse facilitation (PPF)

Researchers have linked PPF to sustained attention (Dawson, Schell, Swerdlow, & Filion, 1997) and orienting attention (Conzelmann et al., 2010). As facilitation occurs when the stimuli are presented with a longer SoA, heart rate slows down and this may indicate waiting for the startling stimulus and guiding attention to this (Graham, 1975). PPF is less well‐understood, in comparison to PPI, which stems from the limited existing research. Studying the neural basis of PPF in both animals and humans will provide more insight into the core neural networks involved in startle modulation.

### 1.3 PPI and PPF: prepulse characteristics, sensory modalities and impact of task instructions

The development of prepulse paradigm protocols applied within current human research have been adapted from animal models studying PPI (Geyer et al., 1990; Swerdlow, Braff, Taaid, & Geyer, 1994). This research uncovered the role of the prepulse in attenuating the startle response in rats by varying parameters of the acoustic prepulse, such as sound intensity, SoA and tone frequency (Hoffman & Searle, 1968; Ison, 1978). This has translated for human research, for example Braff, Geyer and Swerdlow (2001) found greater PPI in both healthy participants and schizophrenia patients when the prepulse presentation was discrete, rather than continuous, and prepulse frequency was white noise, rather than a tone. Investigations into prepulse characteristics has not been conducted for PPF. For PPF, animal and human research show differences in the SoA required to induce PPF. In animals, a much shorter temporal window is required for facilitation, with maximal PPF in hamsters at 100 ms SoA (Sasaki, Iso, Coffey, Inoue & Fukuda, 1998), and mice at <50 ms SoA (Plappert, Pilz & Schnitzler, 2004), rather than 500–6000 ms to facilitate startle reflex response in humans (Putnam & Vanman, 1999).

Prepulse paradigms can also implement different sensory modalities, with the prepulse and pulse stimuli being of the same or different modalities. Typically, an acoustic prepulse paradigm has been used, whereby quiet (prepulse) and loud (pulse) sounds are played. A tactile prepulse paradigm was presented by Swerdlow et al. (2001), administering a weak (prepulse) and a strong (pulse) puff of air. The “fMRI‐friendly” paradigm was used to overcome the logistical difficulties of using auditory stimuli during functional magnetic resonance imaging (fMRI), such as the interference of background acoustic noise from the scanner and its application to clinical populations with hypersensitivity to noise.

PPI and PPF are theorised to reflect automatic processing of sensory information, thus researchers also developed an attention‐to‐prepulse paradigm using sound stimuli. In comparison to no‐task/passive prepulse paradigms, whereby participants are not provided with instructions and passively experience the stimuli, the attention‐to‐prepulse paradigm presents participants with two different prepulses. The two prepulses will usually differ in tone and participants are asked to discriminate between them by paying attention to only one, for example, the higher tone, rather than the lower tone. Employing task instructions applies differential stimulus significance which leads to different allocated attention. It is hypothesised that attending to the prepulse will enhance the inhibitory or facilitatory effect in healthy participants, in comparison to the ignored prepulses (Dawson et al., 1997; Filion, Dawson, & Schell, 1993; Hazlett et al., 1998). Neuroimaging studies have built upon this paradigm to assess the attentional effects on startle modulation (Hazlett et al., 1998, 2001, 2008). In addition, attention‐to‐prepulse paradigms typically use longer sounds as the prepulse, such as “short tone” and “long tone” prepulse sounds which are played for 5–7 s (Filion & Poje, 2003; Thorne, Dawson and Schell, 2005), whereas passive prepulse paradigms use short discrete sounds as the prepulse. Passive prepulse paradigms have demonstrated differential effects of using discrete versus continuous prepulses, with discrete prepulses eliciting greater PPI (Wynn, Dawson, & Schell, 2000), whereas attention‐to‐prepulse paradigms using combinations of discrete and continuous prepulses have demonstrated modulatory effects of prepulse‐to‐pulse intervals (Filion et al., 1993) and PPF (Hazlett et al., 1998).

### 1.4 PPI and PPF: assessment and reliability

Eyeblink is typically used as the physiological measure of startle reflex in humans and can be reliably indexed with electromyography (EMG), whereby electrodes measure orbicularis oculi muscle activity. EMG is a robust measure of startle reflex with high stability when comparing acoustic and tactile stimuli (Kumari, Gray, Geyer, et al., 2003) and high inter‐rater reliability (Heidinger, Reilly, Wang, & Goldman, 2019). A large number of neuroimaging studies using the prepulse paradigm lack the physiological measure of PPI and PPF due to the logistical challenge of implementing EMG within the MRI scanner, meaning that it is difficult to infer modulation of the startle reflex without a simultaneous behavioural measure. However, recent developments have allowed for fMRI compatible EMG (Schulz‐Juergensen, Wunberg, Wolff, Eggert, & Siniatchkin, 2013) or visual monitoring and eye‐tracking methods, such as infra‐red goggles to measure eyeblink during scanning (He et al., 2019; Heidinger et al., 2019). Startle reflex is measured as whole‐body startle in animals, typically in rats (Bubser & Koch, 1994; Geyer, Wilkinson, Humby, & Robbins, 1993) and mice (Ison & Allen, 2007), but eyeblink has also been used as a measure of startle reflex in a few animals. For example, Arnfred, Lind, Hansen and Hemmingsen (2004) used EMG integrated systems to measure PPI in minipigs and demonstrated very consistent findings with humans. However, this research does not exist for PPF.

Modulation of the startle response is measured as a change in eyeblink amplitude or whole‐body response. This change will be present when comparing pulse‐only trials, where the startle response will be at its normal level, to prepulse‐pulse trials, where the response is modulated by the prepulse. Comparing the pulse‐only trials to prepulse‐pulse trials will produce a percentage change, with PPI reflecting a percentage of reduction in magnitude and PPF as a percentage increase in magnitude. The following equation is used:

$$\left[\left(a–b\right)/a\right)]\times 100$$

where *a* = startle amplitude at pulse only trials and *b* = startle amplitude at prepulse‐pulse trials.

The current review will focus on the existing functional neuroimaging research of the startle reflex using prepulse paradigms to induce PPI and/or PPF in human populations, both adults and children. Previous reviews have focussed primarily on PPI (Braff et al., 2001; Geyer et al., 1990; Swerdlow, Geyer, & Braff, 2001), the neural correlates of PPI (Fendt et al., 2001; Swerdlow et al., 2016) and PPI impairments in clinical disorders, namely Schizophrenia (San‐Martin et al., 2020), Schizotypy (Wan, Thomas, Pisipati, Jarvis, & Boutros, 2017), Alzheimer's Disease (Jafari, Kolb, & Mohajerani, 2020) and other psychiatric disorders (Kohl, Heekeren, Klosterkötter, & Kuhn, 2013). There are no current reviews of the PPF literature.

Specifically, this review will provide an overview of the existing functional neuroimaging research using prepulse paradigms by summarising relevant research to outline the neural correlates of prepulse‐elicited startle modulation, and uncover whether the same, overlapping, or different neural networks are employed for PPI and PPF. It will also compare neural activity during PPI and PPF in clinical versus nonclinical populations. Finally, the review will identify the main themes, gaps in knowledge and emerging future directions, including sex differences, maturation of PPI and PPF, translation of the research from animal models, and avenues for future clinical research.

## 2 METHODS

### 2.1 Study selection

**FIGURE 1:**
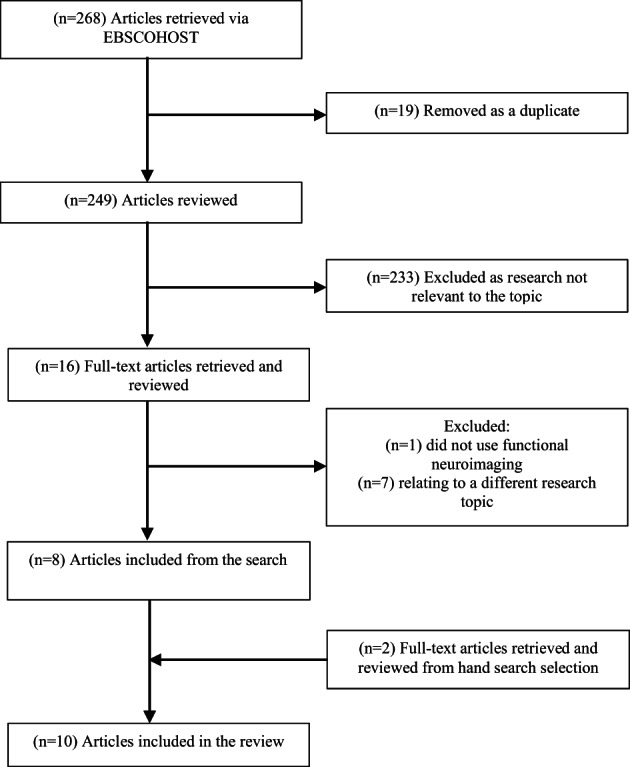
A flowchart of the full search strategy for studies on PPI and PPF

A literature search was conducted using the King's College London EBSCOHOST database (PsycArticles, PsychINFO, Academic Search Complete). The initial search strategy used keywords including: (“prepulse inhibition” OR “PPI”) AND (“fMRI” OR “functional magnetic resonance imaging”). This yielded eight of the papers included in the review. Two papers were hand searched as work cited from a searched article (Hazlett et al., 1998; Kumari, Gray, Geyer, et al., 2003) and included as they fit the inclusion criteria. Additional keywords (“sex differences” OR “gender differences”), (“PET” OR “positron emission tomography”), (“prepulse facilitation” OR “PPF”), were then added, and these produced the hand‐searched papers, but no additional studies were found. Search terms were combined, and field‐codes and wildcards were included, to increase the accuracy of the search. Research articles cited within each of the search results were also screened for relevance and included in the analysis if appropriate. See Figure 1 for the full search strategy.

### 2.2 Data extraction and analysis

Studies were included in the review if they used functional neuroimaging techniques, such as fMRI and PET. Studies were grouped based on participant population, either healthy volunteers or patient populations, and the search uncovered functional neuroimaging research from patient populations in Schizophrenia and TS, only. Studies were also analysed based on prepulse paradigm designs, including type of stimulation (tactile/auditory) and temporal intervals for prepulse‐pulse trials to induce PPI or PPF. This was essential for outlining common parameters across studies and identifying reliable temporal frames for studying PPI and PPF. In addition, study populations were analysed based on sex distribution, in order to review sex differences of PPI and PPF. All functional neuroimaging studies outlined in Table 1.

**TABLE 1:** Summary of neuroimaging studies (in chronological order) of PPI and PPF in healthy and clinical groups

Publication	Sample (% male/ % female)	Design	Imaging modality & approach	Task details PPI/PPF/both sensory modality (acoustic/tactile); pulse and prepulse intensity and duration; prepulse‐to‐pulse intervals (stimulus onset asynchrony, SoA) for PPI/PPF; inter‐trial‐interval (ITI); total number of trials for each trial type)	Psychophysiology data	Behavioural observations	Imaging observations
Hazlett et al. (1998)	Group 1:15 (60/40) Healthy Controls; (mean age = 31.0 years) Group 2:16 (69/31) Schizophrenia Patients, 2 weeks unmedicated to never medicated; (mean age = 37.0 years)	Between‐groups	PET FDG uptake and MRI	Modality: Both (acoustic); Pulse 104 dB, 40 ms; Attended prepulse 1200 Hz, 70 dB, 500 ms or 800 ms; Ignored prepulse 800 Hz, 70 dB, 500 ms or 800 ms; Novel prepulse 500 Hz, 70 dB, 500 ms; SoA: 120 ms, 240 ms, 4500 ms; ITI:29–39 s; No. of Trials: 10 PA, 22 High P (12 PP), 22 Low P (12 PP), 8 Novel P	Offline‐ EMG with electrode at right musculus orbicularis oculi using Ag/AgCl electrodes	Controls: At PP120, greater PPI was observed during attended prepulses, then novel prepulses, then ignored prepulses. No differences were present at PP240. At PP4500, greater PPF was observed during attending prepulses, then novel prepulses, then ignored prepulses. Patients: PPI differed significantly from controls in response to all three prepulse conditions, with patients showing lower PPI responses. At PP240, groups did not differ significantly on PPI. At PP4500, PPF was significantly lower than the control group in response to all prepulse conditions, but overall PPF did not differ significantly.	Controls: Higher rGMR in orbital prefrontal, medial frontal, dorsolateral, and cingulate cortex was observed during PPI when attending to prepulses. When attending to prepulses, a negative correlation between rGMR in superior, middle, and inferior prefrontal cortex was observed and a positive correlation between rGMR in visual association, cortex was observed during PPI at PP120. No neuroimaging findings at PP240 due to lack of attended prepulse pulse induced startle modulation. No neuroimaging findings for PPF presented. Patients: Lower rGMR in bilateral middle and inferior prefrontal cortex and supramarginal, angular and superior parietal lobe during PPI. Greater PPI due to attending to prepulses was negatively correlated with higher rGMR in Brodmann area 10 (left). No neuroimaging findings at PP240 due to lack of attended prepulse pulse induced startle modulation. No neuroimaging findings for PPF presented.
Hazlett et al. (2001)	Group: 10 (50/50) Healthy Controls; (mean age = 27.0 years)	Within‐group	EPI fMRI (ROI)	Modality: PPI (acoustic); Pulse 115 dB, 50 ms, Attended prepulse 1200 Hz, 100 dB, 500 ms or 800 ms, Low prepulse 800 Hz, 100 dB, 500 ms or 800 ms; SoA: 120 ms; ITI: 2, 4, 6 s; No. of Trials: 2 PA, intermixed P and PP trials per block (no. not specified)	None	None	Greater BOLD response during PP trials in right thalamus, left and right anterior nucleus, left mediodorsal nuclei during attended prepulse, rather than ignored. PA trials showed deactivation of these regions. Right anterior cingulate cortex showed similar pattern with greatest BOLD responses during attended prepulses, rather than ignored and a deactivation during PA, but not statistically significant. Left and right BA 32 showed greatest BOLD response during PA trials and smallest BOLD response during PP trials, but this was not significant.
Kumari, Gray, Geyer, et al. (2003)	Group 1:6 (100/0) Healthy Controls; (mean age = 32.5 years) Group 2:7 (100/0) Schizophrenia Patients on antipsychotics; (mean age = 40.0 years)	Between‐groups	EPI fMRI	Modality: PPI (tactile); Pulse 30 psi, 40 ms, Prepulse 10 psi, 20 ms; SoA: 120 ms; ITI: 3–6 s; No. of Trials: Experiment 1 6 PA, Experiment 2 6 PA alternating with PP for each cycle in A/B block design	Offline‐ EMG with electrode at right musculus orbicularis oculi using Ag/AgCl electrodes	Controls: Higher level of PPI, in comparison to patients. Patients: Lower PPI than controls. Startle response on PA trials did not differ between groups.	Controls: PA trials showed deactivations in medial frontal gyrus, pre‐ and post‐central gyrus, middle and superior temporal gyrus, and posterior cingulate. BOLD response in the striatum, thalamus, hippocampus, inferior frontal/middle gyrus and supramarginal gyrus/inferior parietal lobe during PP blocks, relative to PA blocks, and activity in all areas was significantly greater than the patient group. Patients: PA trials showed deactivation only in left middle temporal gyrus. PPI activations seen in controls were not observed in patients, with only greater BOLD response in left post‐central gyrus during PPI. A correlation between PPI and BOLD activity in the CSPT circuitry found for both groups.
Campbell et al. (2007)	Group: 16 (50/50) Healthy Controls; (mean age = 23.0 years)	Within‐group	EPI fMRI (WBA)	Modality: PPI (acoustic); Pulse 110 dB, 50 ms, Prepulse 85 dB, 20 ms; SoA: 120 ms, 480 ms; ITI: 10–12 s; No. of Trials: 20 PA, 20 PP120, 20 PP480	Offline‐ EMG with electrode at left musculus orbicularis oculi using Ag/AgCl electrodes	Significant reduction in mean startle amplitude at PP120, compared to PA. No differences between startle amplitude at PP480, compared to PP120 and PA.	PA trials, compared to PP120, showed increased BOLD in the right medial pons. PP120 trials, compared to PA, showed increased BOLD in the anterior superior frontal gyrus. In comparison to PP480, PP120, showed increased BOLD in the left middle frontal, right superior frontal and right precentral gyrus. PP120 trials, compared to PP480 and PA trials, showed increased BOLD response in the right anterior cingulate, left and right caudate nucleus and pons, and decreased BOLD was observed in the thalamus. PP480 trials showed BOLD increased in the right superior temporal gyrus compared to PP120, whereas the opposite effect occurred with a decrease in the right superior temporal gyrus when comparing PP120 trials to PP480 trials.
Kumari et al. (2007)	Group 1:12 (100/0) Healthy Controls; (mean age = 36.25 years) Group 2:10 (100/0) Schizophrenia Patients medicated (typical antipsychotics); (mean age = 39.0 years) Group 3:10 (100/0) Schizophrenia Patients medicated (risperidone); (mean age = 33.20 years) Group 4:10 (100/0) Schizophrenia Patients medicated (olanzapine); (mean age = 40.20 years)	Between‐groups	EPI fMRI (WBA)	Modality: PPI (tactile); Pulse 30 psi, 40 ms, Prepulse 6 psi, 20 ms; SoA: 30 ms, 120 ms; ITI: 3–6 s; No. of Trials: 5 PA, 5 P, 5 PP30, 5 PP120 per block	Offline‐EMG with electrode at right musculus orbicularis oculi using Ag/AgCl electrodes	Controls: Greater PPI at PP30 and PP120, in comparison to patients. Group 2: Significantly less PPI at PP30 and PP120, in comparison to controls. Group 3: Less PPI at PP30 and PP120 than controls but did not differ significantly, nor differ significantly from Group 2. Group 4: Less PPI at PP30 and PP120 than controls but did not differ significantly; also, not significantly different from Group 2.	Controls: PP30 revealed BOLD activity in left temporal gyrus extending to globus pallidus/putamen, hippocampus and thalamus. During PP120, BOLD activity in the globus pallidus/putamen, caudate, thalamic, insula, inferior frontal, temporal, hippocampal and inferior parietal regions (left) was observed. Thalamic activity and PPI were positively correlated. Group 2: At PP30, no significant activations in but reduced BOLD in anterior and posterior cingulate, right temporal gyrus and thalamus, compared to healthy controls, at PP120. Group 3: Showed similar activity to Group 2, at PP120. Group 4: At PP30, no significant activations in but most resembled the control group, with neural activity in the pre‐ and postcentral gyrus, left middle temporal gyrus extending to the globus pallidus/caudate, hippocampal and thalamic regions at PP120. The strongest BOLD response was observed from the right superior/middle temporal gyrus at PP120.
Hazlett et al. (2008)	Group 1:13 (62/38) Healthy Controls; (mean age = 35.9 years) Group 2:13 (69/31) SPD Patients (unmedicated)^a^; (mean age = 40.1 years) Group 3:13 (77/23) Schizophrenia Patients (unmedicated)^b^; (mean age = 38.5 years)	Between‐groups	EPI fMRI (ROI)	Modality: PPI (acoustic); Pulse 115 dB, 50 ms, Attended prepulse 1,200 Hz, 100 dB, 500 ms or 800 ms, Low prepulse 800 Hz, 100 dB, 500 ms or 800 ms; SoA: 120 ms; ITI: 2, 4, 6 s; No. of Trials: 2 PA, intermixed P and PP trials per block (no. not specified)	None	None	Controls: Greater BOLD response in FST circuitry when attending to prepulses, in comparison to ignored prepulses. Group 2: Did not show FST activity when attending to prepulses but did show greater BOLD response in FST than healthy control when presented with ignored prepulses, rather than attended prepulses. Observed greater BOLD activity in the DLPFC and caudate nucleus during ignored prepulses, rather than attended prepulses, in comparison to healthy controls and schizophrenia patients; and greatest BOLD activation in mediodorsal nucleus, in comparison to healthy controls and schizophrenia patients. Group 3: Did not show FST activity when attending to prepulses but with little change in BOLD activity in FST when presented with attended and ignored prepulses. Observed less differential BOLD in the DLPFC, frontal and temporal regions for attended and ignored prepulses, in comparison to healthy controls and SPD group with diminished BOLD in the DLPFC, caudate nucleus and mediodorsal nucleus of thalamus during attended prepulses, rather than ignored prepulses; and the caudate nucleus and mediodorsal nucleus of the thalamus had the smallest BOLD activation, in comparison to healthy controls and SPD group.
Kumari, Antonova, and Geyer (2008)	Group: 14 (100/0); extended healthy sample from Kumari et al., 2007) Healthy Controls; (mean age = 37.29 years)	Within‐group	EPI fMRI (WBA)	Modality: PPI (tactile); Pulse 30 psi, 40 ms, Prepulse 6 psi, 20 ms; SoA: 30 ms, 120 ms; ITI: 3–6 s; No. of Trials: 5 PA, 5 P, 5 PP30, 5 PP120 per block	Offline‐ EMG with electrode at right musculus orbicularis oculi using Ag/AgCl electrodes	Greater PPI at PP120, compared to PP30. Psychoticism scores negatively correlated with PPI at PP120, PP30 and PA.	PP30 revealed significant left superior temporal gyrus BOLD activity, in comparison to PA. PP120 revealed significant BOLD response in the right inferior parietal cortex extending to the inferior frontal gyrus, in comparison to PA. Psychoticism correlated negatively with neural activity in the inferior parietal lobe, insula, thalamus, and putamen at PP30 and with middle temporal and inferior parietal lobe, insula, putamen, parahippocampus, and fusiform gyrus at PP120.
Neuner et al. (2010)	Group: 15 (100/0) Healthy Controls; (mean age = 28.4 years)	Within‐group	Event‐related fMRI (WBA)	Modality: Both (tactile); Pulse 40 psi, 20 ms, Prepulse 8 psi, 20 ms; SoA: 140 ms, 4,500 ms; ITI: 9.37–28 s; No. of Trials: Section A 6 PA, Section B 18 PA, 18 PP140, 18 PP4500, Section C 6 PA	Online‐EMG with electrode at right musculus orbicularis oculi with custom‐made MR‐compatible EEG cap	PP140 elicited mean percentage change of −37%, whereas at PP4500 elicited mean percentage change of +55%.	In comparison to PA, PP140 trials showed BOLD activity in the right superior parietal lobe and right inferior frontal gyrus, and with correlated EMG measures, this BOLD response was observed in right caudate nucleus, right anterior cingulate cortex, left superior medial gyrus, and right cerebellum. In comparison to PA, PP4500 trials showed BOLD activity in left superior medial gyrus, right middle frontal gyrus, anterior and middle cingulate cortex and cerebellum was observed, and with correlated EMG measures, BOLD response in the right lobule VIII and left lobule X were observed. A conjunction analysis of PA and PP trials revealed somatosensory cortices, right thalamus and right insula, and with correlated EMG measures, the left middle orbital gyrus and left thalamus.
Zebardast et al. (2013)	Group 1:20 (60/40) Healthy Controls; (mean age = 30.4 years) Group 2:18 (61/39) TS Patients; (mean age = 33.2 years)^c^	Between‐groups	EPI fMRI (WBA)	Modality: PPI (tactile); Pulse 80 psi, 40 ms, Prepulse 7 psi, 20 ms; SoA: 120 ms; ITI: 3–6 s; No. of Trials: 6 PA, 6 PP	Online‐ EMG with electrode at left musculus orbicularis oculi	Controls: normal levels of PPI observed at PP120. Patients: PPI was significantly reduced in TS patients, in comparison to healthy controls.	Controls: Increased BOLD in frontal gyrus, superior middle and inferior middle temporal gyrus, paracentral lobule, occipital gyrus, insula, anterior and posterior cingulate, parahippocampal gyrus and corpus callosum during PP, in comparison to PA. There was a positive correlation between PPI and left middle frontal gyrus for the control group. Patients: Reduced BOLD in the orbitofrontal cortex, lateral frontal cortex, anterior insula, posterior cingulate cortex, middle temporal gyrus, left and right caudate, and posterior cerebellum during PP, in comparison to PA. No correlation present between PP and left middle frontal gyrus. There was a positive correlation between tic severity and BOLD response in the left caudate, and a positive correlation between severity of obsessive–compulsive symptoms and BOLD response in the left orbito/inferior frontal cortex during PP, in comparison to PA.
Buse et al. (2016)	Group 1:22 (100/0) Healthy Controls; (mean age = 14.14 years) Group 2:9 (100/0) TS Patients, all on medication^d^; (mean age = 13.45 years)	Between‐groups	Event‐related fMRI (WBA)	Modality: PPI (tactile); Pulse 40 psi, 40 ms, Prepulse 6 psi, 20 ms; SoA: 140 ms; ITI: 3–10s; No. of Trials: 40 PA, 40 PP	Online‐EMG with electrode at left musculus orbicularis oculi and at electrode FP1 using fMRI compatible Ag/AgCl electrodes	Controls: A significant reduction of startle amplitude at PP140, compared to PA. PPI was higher, in comparison to patients. Patients: No significant difference between startle amplitude at PA and PP140, indicating no PPI.	Controls: PPI BOLD activity during PP trials, compared to PA, was observed in the medial and inferior frontal gyrus, premotor gyrus, post‐central gyrus, precuneus, middle occipital gyrus, insula, cingulate gyrus and thalamus. Patients: Less BOLD activity in PP140 trials, compared to PA, in middle frontal gyrus, postcentral gyrus, superior parietal cortex/precuneus, cingulate gyrus and caudate body, compared to control.

Abbreviations: BOLD, blood oxygen level dependent; EMG, electromyography; EPI, Echo Planar Imaging; FDG, F‐fluoro‐deoxyglucose; ITI, inter‐trial interval; fMRI, functional magnetic resonance imaging; MRI, magnetic resonance imaging; P, prepulse only condition; PA, pulse alone condition; PET, Positron emission tomography; PP, prepulse‐pulse condition; PPI, prepulse inhibition; rGMR, relative glucose metabolic rate; ROI, regions of interest; SEM, startle eyeblink modification; TS, Tourette syndrome; WBA, whole brain analysis.

^a^ 12 = never medicated, 1 = once received antipsychotics 10 years prior to study.

^b^ 5 = never medicated, 8 = previously on psychoactive medication but off up to 2 weeks prior to study.

^c^ 5.9% = Alpha‐2 agonists, 11.8% = neuroleptics, 41.2% = selective serotonin reuptake inhibitors, 17.6% = benzodiazepines.

^d^ 5 = tiapride, 1 = aripiprazole, 1 = aripiprazole and fluoxetine, 1 = pimozide, 1 = risperidone.

## 3 RESULTS

The systematic search yielded 10 studies, with nine studies using fMRI and one study using PET, to study PPI alone (nine studies) or PPI and PPF (one study) in healthy participants and/or patient groups. Of the PPI studies, three studies used healthy participants, three studies used schizophrenia patients with a healthy control group, one study used schizophrenia patients with two control groups (healthy participants and participants with schizotypy), and two studies used participants with TS and a healthy control group. The PPI and PPF study used healthy participants. The findings of all reviewed PPI/PPF studies are presented in Table 1 and described in the Section 3.

### 3.1 Neuroimaging studies in healthy adults

Mapping neural networks in healthy participants allows researchers to develop a blueprint for normal functioning cognition and behaviour. In addition, it provides a baseline against which to evaluate neural abnormalities, both structural and functional, which may underlie cognitive deficits in a number of clinical populations.

#### 3.1.1 PPI

Early research using PET indicated a “top‐down” influence for processing prepulse stimuli in humans, implicating cortical and limbic regions in modulating the startle response (Hazlett et al., 1998). Hazlett et al. (1998) designed an attention‐to‐prepulse paradigm, whereby participants were presented with a pulse and three prepulses; a higher tone to attend to, a lower tone to ignore, and a novel prepulse. In healthy controls at 120 ms SoA, attended prepulses showed greater PPI, in comparison to ignored and novel prepulses, but novel prepulses had a greater inhibitory effect than ignored prepulses, although this effect was not seen on 240 ms SoA trials. A negative correlation was observed between glucose metabolism in the prefrontal cortex and PPI (120 ms SoA), with greater PPI reflecting greater relative glucose metabolism in these neural regions. Building upon this work, fMRI research has highlighted thalamic involvement during PPI in healthy participants. Hazlett et al. (2001) used an attention‐to‐prepulse paradigm, whereby participants attended to higher tone prepulses and ignored lower tone prepulses that were presented 120 ms SoA. Greater BOLD response was seen in the thalamus, anterior nucleus and mediodorsal nucleus when participants attended to the prepulse, rather than to the ignored prepulse, and deactivation of these neural regions occurred during pulse‐only trials.

Using a tactile prepulse paradigm, Kumari, Gray, Geyer, et al. (2003) mapped neural activity in healthy volunteers, as a control group for the participants with schizophrenia, when participants passively experienced the tactile prepulse and pulse stimuli. The healthy control group, compared to participants with schizophrenia, displayed significantly greater BOLD activity in the striatum, thalamus, hippocampus, inferior frontal/middle gyrus and supramarginal gyrus/inferior parietal lobe during 120 ms SoA, when compared to pulse‐only trials. PPI trials (120 ms SoA) and BOLD activity in the CSPT circuitry was positively correlated in healthy controls.

Kumari et al. (2007) explored the neural correlates of PPI in healthy controls and medicated participants with schizophrenia. Implementing the tactile prepulse paradigm from Kumari, Gray, Geyer, et al. (2003), with PPI induced using multiple prepulse‐to‐pulse intervals (30 and 120 ms SoA). Healthy controls showed BOLD activity in the globus pallidus/putamen, caudate, thalamic, insula, inferior frontal, temporal, hippocampal and inferior parietal regions at 120 ms SoA, compared to pulse‐only trials. During 30 ms SoA trials, there was also increased BOLD response in the temporal gyrus, extending to striatal, thalamic and hippocampal regions seen active on 120 ms SoA trials.

In Campbell et al.'s (2007) study, healthy adult participants were presented with prepulse (120 and 480 ms SoA) and pulse sounds whilst watching a silent film. Increased BOLD response in the middle frontal gyrus, superior frontal gyrus and precentral gyrus was noted when comparing prepulse‐pulse trials of 120 to 480 ms SoA. A significant increase in BOLD activity was reported in the superior temporal gyrus for 480 ms SoA trials, in comparison to a decreased BOLD response for 120 ms SoA trials. EMG‐fMRI identified PPI‐related BOLD activity in the anterior cingulate, caudate nuclei and pons and a decreased BOLD response in the thalamus, in comparison to 480 ms SoA trials and pulse‐only trials. Campbell et al. (2007) outlined the role of frontal regions in PPI, including the superior and middle frontal gyrus activation at 120 ms SoA, and increased BOLD response in the anterior cingulate during 120 ms SoA, in comparison to reduced activation during pulse‐only and 480 ms SoA.

Kumari et al. (2008) employed tactile stimuli to investigate the association between psychosis‐proneness and neural activity of PPI in healthy adult participants. As PPI impairments have been observed in patients with schizophrenia, this study explored whether PPI was impaired in healthy participants who scored high in psychosis‐proneness, measured with Eysenck Personality Questionnaire Revised (EPQ‐R, Eysenck & Eysenck, 1998). It was found that psychoticism correlated negatively with PPI on prepulse‐pulse trials (30 and 120 ms SoA). On 30 ms SoA trials, participants showed increased superior temporal gyrus BOLD activity, in comparison to pulse‐only trials, whereas on 120 ms SoA trials, increased BOLD activity in the inferior parietal cortex extending to the inferior frontal gyrus was observed, in comparison to pulse‐only trials. Psychoticism correlated negatively with neural activity in the inferior parietal lobe, insula, thalamus and putamen on 30 ms SoA trials, and with the middle temporal lobe, inferior parietal lobe, insula, putamen, parahippocampus, and fusiform gyrus during 120 ms SoA trials.

Using the attention‐to‐prepulse paradigm with acoustic stimuli, Hazlett et al. (2008) used fMRI to study the frontal‐striatal‐thalamic (FST) circuitry, comparing a healthy control group with a group of unmedicated schizophrenia participants and participants with Schizotypal Personality Disorder (SPD). Hazlett et al. (2008) found that healthy controls exhibited greater BOLD response in the FST circuitry when attending to prepulses, in comparison to the ignored prepulses on 120 ms SoA trials.

Zebardast et al. (2013) studied the neural correlates of tactile PPI in healthy adults, compared to a group of adults with TS. Nine distinct brain regions were related to PPI trials and healthy controls exhibited greater neural activity during the 120 ms SoA trials, compared to pulse‐only trials. These regions included: the orbitofrontal cortex, lateral frontal cortex, anterior insula, posterior cingulate cortex, middle temporal gyrus, caudate and posterior cerebellum.

Buse et al. (2016) implemented EMG‐fMRI to study PPI in boys (11–17 years), comparing a healthy control group with a matched group of boys diagnosed with TS. Using a younger population would remove the effects of medication and plasticity, and a male population would remove the confound of sex differences, which have been reported with developing TS (see Leckman, 2002; Leckman et al., 1998). Using a similar tactile study design to Zebardast et al. (2013) but using PPI trials of 140 ms SoA, which in the context of prepulse‐to‐pulse intervals is a deviation from most studies when producing an inhibitory effect in the eyeblink response, the control group demonstrated greater BOLD activity than the clinical group at 140 ms SoA, in comparison to pulse‐only trials, in the medial and inferior frontal gyrus, premotor gyrus, post‐central gyrus, precuneus, middle occipital gyrus, insula, cingulate gyrus and thalamus.

#### 3.1.2 PPF

Only one study has reported the neural correlates of both PPI and PPF, as the only other neuroimaging study to have included PPF as a condition did not provide analyses or findings (Hazlett et al., 1998). Neuner et al. (2010) presented healthy male participants with puffs of air as the prepulse and pulse, with the prepulse either preceding the pulse by 140 ms to elicit PPI, or 4500 ms to elicit PPF. The 140 ms SoA trials, in comparison to pulse‐only trials, showed BOLD activity in the superior parietal lobe and inferior frontal gyrus. When correlated with the EMG measures, BOLD response was observed in the caudate nucleus, anterior cingulate cortex, superior medial gyrus and cerebellum. During the 4500 ms SoA trials, BOLD activity in the superior medial gyrus, middle frontal gyrus, anterior and middle cingulate cortex and cerebellum was observed, in comparison to pulse‐only trials. When correlated with the EMG measures, lobule VIII and lobule X were active, in comparison to pulse‐only trials. Moreover, the conjunction analysis for pulse‐only, PPI trials and PPF trials indicated activity of somatosensory cortices, thalamus, insula and middle orbital gyrus. Neuner et al. (2010) proposed this common neural network for PPI and PPF, with the overlap of neural activity in the frontal lobe, anterior/middle cingulate cortex and cerebellum.

#### 3.1.3 Summary of neuroimaging findings

Thalamic activity during PPI conditions was present in six of 10 studies of PPI in healthy groups. The role of the thalamus is evident in mediating the startle reflex and producing an inhibitory response in healthy adult populations (Kumari, Gray, Geyer, et al., 2003; Kumari, et al., 2007, 2008; Buse et al., 2016). Comparing attention‐to‐prepulse paradigms with passive prepulse paradigms, it is evident that there is an attentional effect of the thalamus, with greater BOLD response in thalamic regions is observed when participants attend to prepulses, rather than ignoring prepulses (Hazlett et al., 2001, 2008). On both paradigm types, pulse‐only trials, where startle reflex is not modulated, compared to PPI trials, show a decrease in thalamic BOLD activity, further illustrating its role in normal functioning startle reflex and, therefore, sensorimotor gating.

The role of the striatum during PPI conditions was present in five of 10 studies of PPI in healthy groups (Campbell et al., 2007; Hazlett et al., 2008; Kumari et al., 2007; Kumari, Gray, Geyer, et al., 2003; Neuner et al., 2010). Frontal cortex activity has been documented in eight of 10 studies, demonstrating how higher order neural regions are recruited to modulate the startle reflex. This activity is evident during prepulse‐pulse trials at 120 ms (Hazlett et al., 1998, 2008; Kumari, Gray, Geyer, et al., 2003; Kumari et al., 2007; Campbell et al., 2007; Zebardast et al., 2013), 140 ms SoA (Buse et al., 2016; Neuner et al., 2010), in comparison to pulse‐only trials which elicit a brainstem response. Consistently, 30 ms SoA trials show increased BOLD response in the superior temporal gyrus in healthy adults (Kumari et al., 2007, 2008). This has similarly been seen on 480 ms SoA trials, with decreased BOLD response in the superior temporal gyrus in 120 ms SoA trials (Campbell et al., 2007). The neuroimaging evidence from healthy adult populations clearly and consistently outlines the CSPT circuitry involved in PPI, extending from the brain stem to incorporate limbic and frontal regions. With this evidence and a wealth of consistent PPI neural findings, it is clear that this research becomes a basis for understanding PPI deficits in clinical disorders, and this is further supported by Kumari et al. (2008) findings highlighting the relationship between psychosis‐proneness and CSPT circuitry.

PPF neuroimaging findings suggest neural networks implementing the frontal cortex and cerebellum, but this is yet to be replicated in existing functional neuroimaging studies. The literature suggests a neural network of startle reflex and modulation, combining pulse‐only, PPI and PPF trials, to implement the somatosensory cortices, thalamus, insula, middle orbital gyrus, middle cingulate cortex and cerebellum in a circuit of activity from startle reflex to modulation. These neuroimaging findings from healthy adult populations pave the way for further investigations of PPF in order to create a replicable and reliable base for further clinical research.

### 3.2 Neuroimaging studies: clinical populations

This literature search yielded functional neuroimaging research in clinical populations using prepulse paradigms only in two clinical populations: schizophrenia and TS.

#### 3.2.1 Schizophrenia

In addition to those affected by the schizophrenia (see Section 1), PPI is also reduced in unaffected biological relatives of schizophrenia patients (Cadenhead, Geyer, & Braff, 1993; Kumari et al., 2005) and on the spectrum of schizophrenia disorders, including schizotypal disorder (see Li, Du, Li, Wu, & Wu, 2009; Wong & van Tol, 2003) and psychosis‐proneness (Kumari et al., 2008). Gottesman and Gould (2003) posit that PPI may be an endophenotype of schizophrenia, highlighting a clear genetic connection. Quednow et al.'s (2018) meta‐analysis found strong associations between PPI and gene polymorphisms, specifically catechol‐O‐methyltransferase (*COMT, rs4680*), glutamate receptor ionotropic kainite 3 (*GRIK3, rs1027599*), transcription factor 4 (*TCF4, rs9960767*) and proline dehydrogenase (*PRODH, rs385440*), demonstrating the role of these gene variations in developing information processing deficits. PPF may also be reduced in schizophrenia patients, in comparison to healthy controls (Kumari, Aasen, & Sharma, 2004).

##### PPI

Using the attention‐to‐prepulse paradigm, Hazlett et al. (1998) found that participants with schizophrenia did not show differential PPI between the attended and ignored prepulse at 120 and at 240 ms SoA, the latter being the same findings as the control group. The clinical group showed lower glucose metabolism during PPI at 120 ms SoA in the superior, middle and inferior prefrontal cortex and supramarginal, angular and superior parietal lobe, in comparison to the control group. PPI was also negatively correlated with higher glucose metabolism in the anterior prefrontal cortex (Brodmann area 10) when participants with schizophrenia were presented with the attended prepulses.

Comparing a healthy control group with participants diagnosed with schizophrenia, this tactile prepulse paradigm design by Kumari, Gray, Geyer, et al. (2003) highlighted that PPI in the clinical group was lower, compared to the controls. There was significantly less BOLD activity in striatal, thalamic, hippocampal, frontal and supramarginal gyrus/inferior parietal lobe during 120 ms SoA when compared to pulse‐only trials in the clinical group, as compared to healthy controls. A linear relationship was also found between PPI and BOLD activity in the CSPT circuitry in participants with schizophrenia, which corresponded with the level of observed PPI.

Kumari et al. (2007) explored the neural correlates in a group of medicated schizophrenia participants (typical and atypical antipsychotics), and a healthy control group. The study reported lower PPI in the clinical group, in comparison to healthy controls, and also less PPI in participants taking typical antipsychotics, in comparison to healthy controls. Participants on atypical antipsychotics (risperidone and olanzapine) also showed this, but not to a statistically significant level. Patients showed reduced BOLD response in the temporal lobe, thalamic and striatal regions on 120 ms SoA trials, compared to pulse‐only trials. Thalamic activity was significantly associated with PPI across all clinical groups. Differences between the clinical groups based on medication type showed reduced BOLD response at 120 ms SoA in the anterior and posterior cingulate, right temporal gyrus and thalamus in the typical antipsychotic group, compared to healthy controls. This was similarly seen in participants taking risperidone. Interestingly, participants taking olanzapine most resembled the neural activity exhibited by the control group at 120 ms SoA, with neural activations in the pre‐ and postcentral gyrus, left middle temporal gyrus, extending to the globus pallidus/caudate, hippocampal and thalamic regions. The strongest BOLD response was observed in the right superior/middle temporal gyrus. PPI differences are still present in the medicated schizophrenia groups, compared to controls, but the reduction of PPI is less pronounced in the atypical medication groups, highlighting a possibility of “greater PPI‐improving effects” (Kumari et al., 2007, pg. 471).

Findings by Hazlett et al. (2008) using the attention‐to‐prepulse paradigm outlined that unmedicated participants diagnosed with schizophrenia did not show changes in BOLD activity in response to attended and ignored prepulses in FST circuitry. Moreover, participants showed less differential BOLD activity in the dorsolateral prefrontal cortex (DLPFC), frontal and temporal regions for attended and ignored prepulses, in comparison to healthy controls and the SPD group; and the caudate nucleus and mediodorsal nucleus of the thalamus had the smallest BOLD activation, in comparison to healthy controls and the SPD group. On the other hand, participants with SPD showed greater BOLD response in FST circuitry during ignored prepulses, rather than attended prepulses, showing an abnormal BOLD attentional response compared to controls. The SPD group also showed greater BOLD activity in the DLPFC and caudate nucleus in response to ignored prepulses, rather than attended prepulses, in comparison to controls and schizophrenia patients; and showed greatest BOLD activation in mediodorsal nucleus, in comparison to healthy controls and schizophrenia patients. In summary, diminished BOLD activity was noted in the DLPFC, caudate nucleus and mediodorsal nucleus of thalamus when participants with schizophrenia attended to the high tone prepulses, whereas the SPD group demonstrated the opposite effect with greater BOLD response in these regions when ignoring low tone prepulses, in comparison to healthy controls.

Overall, functional abnormalities in superior, middle and inferior prefrontal cortices have been highlighted in schizophrenia using the attention‐to‐prepulse paradigm. Participants with schizophrenia demonstrated impairments in the attentional aspect of discriminating between prepulses, indicating that they did not have attentional resources to evaluate the prepulses. However, PPI was positively correlated with anterior prefrontal cortex activity when attending to prepulses, rather than in relation to ignored and novel prepulses. Less differential BOLD in the DLPFC, frontal and temporal regions was observed for attended and ignored prepulses, compared to healthy controls and participants with SPD. Using passive prepulse paradigms, participants with schizophrenia have demonstrated significantly lower BOLD response in striatal, thalamic, hippocampus, inferior frontal/middle gyrus, temporal lobe and supramarginal gyrus/inferior parietal lobe on 120 ms SoA trials, in comparison to healthy controls. CSPT BOLD activity was positively correlated with PPI in both schizophrenia and healthy populations. Medication type in schizophrenia also produced differential PPI neural findings on 120 ms SoA trials, with participants taking typical antipsychotic and participants taking risperidone showing less BOLD activity in temporal lobe, thalamic and striatal regions, compared to healthy controls. However, participants taking olanzapine showed similar neural findings to healthy controls with strong BOLD response PPI‐related neural regions.

##### PPF

PPF deficits have been noted in participants with schizophrenia during offline studies, with generally lower PPF with 1000–4500 ms intervals in participants with schizophrenia, compared to healthy controls (for a review of behavioural findings, see Kumari et al., 2004). Early research from Hazlett et al. (1998) using the attention‐to‐prepulse paradigm demonstrated differences in PPF between healthy controls and a group of unmedicated schizophrenia participants, with healthy controls showing more PPF when attending to prepulses at 4500 ms SoA. The clinical group failed to show this differential effect between attended and ignored prepulses, but did show more PPF during novel prepulses, rather than ignored. However, neural findings accompanying these behavioural effects were not presented by these authors.

#### 3.2.2 Tourette syndrome

TS is characterised as a childhood neuropsychiatric disorder and is associated with sensorimotor gating deficits, such as premonitory urges and motor and phonetic tics which result in impairments in gating sensory, cognitive and motor information (Leckman, 2002). Age of onset is typically prepubertal and it is more common in boys, than girls. TS is less common in adulthood with reduced severity in symptoms by the age of 20 years (Bloch et al., 2006; Leckman et al., 1998). Due to the nature of the symptoms of TS, it is important to understand abnormalities in the integration of sensory and motor systems. Sowell et al. (2008) identified thinning of somatosensory cortices and connectivity between motor and sensory cortices in participants with TS, illustrating structural differences to healthy controls. Functional neuroimaging research has identified both striatal regions and the hippocampus as playing a key role in TS (Bloch, Leckman, Zhu, & Peterson, 2005; Kalanithi et al., 2005) and, as these regions form part of the CSPT network, it indeed may be that this pathophysiology may lead to sensorimotor disturbances in TS. Reduced PPI has been observed in TS populations, in comparison to healthy controls, but PPF has yet to be studied in a TS population.

##### PPI

The “fMRI‐friendly” startle paradigm by Swerdlow, Karban, et al. (2001), was developed and studied by presenting children with TS with acoustic and tactile prepulses and pulses. The clinical group showed reduced PPI at 120 ms SoA, in comparison to the control, when presented with both modalities. Acoustic PPI was greater than the tactile PPI in both groups, but the general consensus was that PPI impairments can be explored using both stimulus modalities. The “fMRI‐friendly” tactile design seems to dominate the existing literature for TS populations, with no current studies to investigate the neural correlates of PPI in TS using auditory stimulation.

Zebardast et al. (2013) outlined nine distinct PPI‐related brain regions, namely the orbitofrontal cortex, lateral frontal cortex, anterior insula, posterior cingulate cortex, middle temporal gyrus, left and right caudate, and posterior cerebellum. The clinical group demonstrated lower PPI than the control group at 120 ms SoA and less BOLD activity in these regions. A linear relationship was observed between PPI and the left middle frontal gyrus activation for the control group, but this was not present for the patient group. Interestingly for the clinical group, there was a significant positive correlation between tic severity and BOLD activity in the caudate during PPI. Moreover, there was a significant positive correlation between the severity of obsessive–compulsive symptoms in participants with TS and activity in the orbito/inferior frontal cortex. Gender, age, co‐morbidities (of OCD and Attention Deficit Hyperactivity Disorder [ADHD]), and medication in participants with TS did not show any effect on these findings.

Buse et al. (2016) found that in participants with TS, reduced BOLD response was observed in the middle frontal gyrus, postcentral gyrus, superior parietal cortex, cingulate gyrus and caudate on 140 ms SoA trials, compared to healthy controls.

Neuroimaging findings from participants with TS outline significant differences in BOLD activity in comparison to a control group, with reduced BOLD response when PPI trials are compared to pulse‐only trials, in the orbitofrontal cortex, lateral frontal cortex, middle frontal gyrus, postcentral gyrus, superior/parietal cortex/precuneus, anterior insula, posterior cingulate cortex, middle temporal gyrus, caudate and posterior cerebellum (Buse et al., 2016; Zebardast et al., 2013). In addition, there was a positive correlation between tic severity and BOLD response in the caudate during PPI trials, and severity of obsessive–compulsive symptoms and BOLD response in the orbito/inferior frontal cortex, demonstrating this neural activity may be a biomarker of these symptoms. It is evident that these neural regions form an essential part of the neural network which have central roles in integrating sensory information, meaning that a dysfunction in these areas highlight an abnormality in sensory information processing in TS, causing PPI impairments.

## 4 DISCUSSION

### 4.1 PPI and PPF neural circuitry: a shared or separate neural network?

From the wealth of literature, it is clear that PPI provides a robust measure of sensorimotor gating with consistent mapping of normal functioning neural circuitry involved in PPI, namely thalamic, striatal and frontal lobe regions which mediate the startle reflex in the brainstem (see Section 3.1.1). These functional neuroimaging findings are also in line with those from structural MRI studies, which showed a positive association between PPI and grey matter volumes in most of these regions (Kumari et al., 2005), and with PPI and superior parietal cortex and frontal cortex (Hammer et al., 2013). Neuroimaging findings at peak PPI, 120 ms SOA in both acoustic and tactile stimulation (Campbell et al., 2007; Hazlett et al., 2008; Kumari et al., 2007), reveal the recruitment of higher order control from the CSPT neural circuitry to mediate the automatic brainstem response. The neurobiology of PPI at longer/peak lead interval PPI appears to overlap with that of the P50 suppression (Oranje, Geyer, Bocker, Kenemans, & Verbaten, 2006). P50 suppression (i.e., a smaller P50 evoked potential to the second stimulus when two sensory stimuli are presented by a 500 ms interval) is another paradigm used widely to document inhibitory deficits in people with schizophrenia (Patterson et al., 2008).

PPF‐related neural activity in the left superior medial gyrus, right middle frontal gyrus, anterior and middle cingulate cortex and cerebellum has been identified in the only fMRI study of PPF (Neuner et al., 2010). PET research by Hazlett et al. (1998) presented PPI and PPF behavioural findings from healthy controls and schizophrenia patients, but only detailed PPI‐related neural activity at 120 ms, rather than PPF‐related neural activity at 4500 ms.

It is clear that there has been little exploration into whether startle modulation (inhibition and facilitation of the startle reflex) operate on one shared neural circuit or two separate pathways. Neuner et al. (2010) suggested a shared neural network for startle modulation, with activity for facilitation and inhibition occurring in the frontal lobe, anterior/middle cingulate cortex and cerebellum. Establishing the neural correlates of PPI and PPF is essential for further understanding normal and aberrant cognitive functioning relating to sensorimotor gating and attentional processing and how the two functions interact.

### 4.2 Neural findings from passive versus attention‐to‐pulse paradigm

In healthy control groups, similar PPI and PPF have been reported in attention‐to‐prepulse paradigms (Dawson et al., 1997; Hazlett et al., 1998, 2001, 2008) and passive prepulse paradigms (Campbell et al., 2007; Kumari et al., 2008; Neuner et al., 2010), demonstrating an increase in modulatory effect due to prepulse‐pulse pairing. In addition, neural findings for PPI from the attention‐to‐prepulse paradigms (Hazlett et al., 1998, 2001, 2008) have mapped thalamic activity which is also seen using passive prepulse paradigms (Campbell et al., 2007; Kumari et al., 2008; Neuner et al., 2010).

## 5 FUTURE DIRECTIONS

### 5.1 Mapping neural correlates of PPI and PPF

One of the major gaps in the field is mapping the neural correlates of PPF and establishing whether there is one shared or two separate neural pathways for PPI and PPF. In animal research, it is assumed that PPI and PPF demonstrate independent processes due to the different effect produced by the prepulse, such as prepulse intensity and SoA (Plappert et al., 2004). Kumari, Gray, Gupta, et al. (2003) provides behavioural evidence for an overlap of neural circuitry with some distinct areas involved in the underlying process of PPI and PPF as women showed a smooth transition from PPI to PPF, indicating a general shift from PPI to PPF on a shared neural network. However, this was not seen in men. In addition, fMRI research has suggested an overlap in neural networks for PPI and PPF (Neuner et al., 2010). PPF requires more investigation to clarify the core networks of startle modulation. If human research has identified an overlap and single underlying process which PPI and PPF operate on, does this mean that PPF deficits may also be present in these clinical disorders? This leaves a huge gap in our understanding of startle modulation and how this may benefit clinical populations.

Future functional neuroimaging research should ensure that PPI and PPF conditions are separate and discrete, rather than interweaving conditions, as this will provide clear interpretations of related BOLD response to identify the neural networks of PPI and PPF. It may also be advantageous to employ both auditory and tactile stimuli in the prepulse paradigms. For example, implementing technological advances to overcome the constraints of background noise in conventional fMRI, such as silent fMRI (Damestani et al., 2021; Wiesinger, Menini, & Solana, 2019), will allow for auditory stimulation and neural responses resulting from this stimulation, rather than scanning conditions, or also to build upon the earlier interventions of developing an “fMRI‐friendly” prepulse paradigm (Swerdlow, Karban, et al., 2001).

### 5.2 Understanding sexual dimorphism in PPI and PPF

At present, it is unclear how sex can affect the underlying neural substrates of startle modulation using the prepulse paradigm. Sex differences have been identified across the literature in both humans and animals (Kumari, 2011), with women demonstrating less PPI than men (Aasen, Kolli, & Kumari, 2005; Abel, Waikar, Pedro, Hemsley, & Geyer, 1998; Kumari et al., 2004), even after removing confounds such as personality characteristics attributed to the men and women (Kumari, Gray, Gupta, et al., 2003), and smoking (Aasen et al., 2005). Sex differences in PPI may result from reproductive hormones. For example, post‐menopausal women and pre‐pubescent girls (>8 years) do not show reduced PPI, in comparison to men (Kumari et al., 2008; Ornitz, Guthrie, Sadeghpour, & Sugiyama, 1991), and women in the early follicular stage of their menstrual cycle, show more PPI than women in the luteal phase (Kumari et al., 2010). Women in their third trimester of pregnancy display low PPI compared to postpartum women, linked to higher than normal hormone levels (Kask, Bäckström, Gulinello, & Sundström‐Poromaa, 2008). Alternatively, PPF is higher in women than men, including post‐menopausal women and pre‐menopausal women, in comparison to men, and also higher in pre‐pubescent girls (>8 years) compared to prepubescent boys (>8 years) (Kumari et al., 2008; Kumari, Gray, Gupta, et al., 2003; Ornitz et al., 1991). Women in the early follicular stage of their menstrual cycle show less PPF than women in the luteal phase (Kumari et al., 2010).

Sexual dimorphism in startle modulation may also be a result of the combination of hormones on the neural system. For example, oestrogen influences dopaminergic activity in the nucleus accumbens and striatum (Becker, 2000), which are essential in inhibiting the startle response. Progesterone is also involved in dopaminergic activity by increasing and decreasing basal and amphetamine‐stimulated dopamine and cholinergic activity, which plays a role in PPI in humans and animals (Geyer et al., 2001; Rupprecht et al., 1999).

Sex differences have been noted in a number of clinical disorders, including schizophrenia and TS. For example, in schizophrenia, there are less severe cases, later age of onset and often better outcomes for women with schizophrenia, rather than men with schizophrenia (see Leung, Chue, & Psych, 2000; McGrath et al., 2004), and TS is more prevalent in males than females (see Leckman et al., 1998; 2002). Both disorders show widely documented PPI deficits, suggesting that outlining the effect of sex on PPI‐related neural substrates will explain the sexual dimorphism of these clinical disorders.

### 5.3 Maturation of PPI and PPF in children

The majority of literature discussed has studied adult populations, with the exception of Buse et al. (2016). Using PPI and PPF as biomarkers for clinical research also suggests that investigation of the maturation of startle modulation in children could indicate early development of disorders with PPI and PPF impairments. Research has revealed no significant startle modulation in infants (2–6 months) and toddlers (15 months) (Balaban, Anthony, & Graham, 1989; Graham, Strock, & Zeigler, 1981) but from 3 to 10 years, children demonstrate increasing maturation for PPI, reflecting adult levels of peak inhibition at 120 ms SoA by 9 years, and adult levels of inhibition at 60 ms SoA by 10 years (Gebhardt, Schulz‐Juergensen, & Eggert, 2012). Earlier research depicted this age to be at 8 years (Ornitz, Guthrie, Kaplan, Lane, & Norman, 1986), thus it can be inferred that PPI matures to adult level between 8 and 10 years. PPF has been reported at 2000 and 4500 ms SoA in children, similarly to adults (Hawk Jr, Pelham Jr, & Yartz, 2002), with maturation of PPF at 8 years (Ornitz et al., 1986). To our current knowledge, there are no neuroimaging studies to map neural activity in healthy children during PPI or PPF. In addition, clinical disorders with PPI and PPF deficits have been identified in a number of childhood disorders, namely TS (Buse et al., 2016) and enuresis (Ornitz, Hanna, & de Traversay, 1992).

### 5.4 Longitudinal research: a study of normal or aberrant development

To expand the field, researchers may wish to conduct longitudinal research, implementing functional neuroimaging techniques to compliment the changes in development of PPI and PPF, as seen from pre‐ to post‐puberty. Indeed, observing these changes in a population where hormonal change occurs in puberty will allow us to study the effect of hormones and sexual dimorphism on PPI and PPF (Abel et al., 1998; Kumari, Gray, Gupta, et al., 2003, Kumari et al., 2004; Aasen et al., 2005; Kumari, 2008, 2010, 2011).

Moreover, this would allow researchers to ask, “at what point is aberrant development of PPI and PPF seen and what are its consequences in terms of mental health and functioning?” These neural changes, seen in a developing cohort of children, would indicate, for example, if psychosis does or does not develop, and why. Specifically exploring more high‐risk populations would allow for observation of whether normal maturation of PPI and PPF occurs, such as unaffected relatives of psychosis patients (Cadenhead, Swerdlow, Shafer, Diaz, & Braff, 2000; Kumari et al., 2005), and individuals with gene polymorphisms with variations associated with lower PPI (Quednow et al., 2018). Therefore, assessing a cohort who are clinically high‐risk for developing psychosis‐spectrum disorders would advance the field, assessing PPI and PPF with complimentary neural findings.

In addition to psychosis‐spectrum disorders, longitudinal studies can explore high‐risk clinical groups and the pre‐manifestation of disorders, such as Huntington's disease, that show sensorimotor gating disturbances (Uc, Skinner, Rodnitzky, & Garcia‐Rill, 2003). For example, the pre‐diagnostic stage of Huntington's disease shows no detectable clinical abnormalities (see Walker, 2007), thus PPI could be used as a biomarker and a marker of clinical severity (de Tommaso et al., 2001), with complimentary neuroimaging findings to explore where neural function also shows deficit.

### 5.5 Translation of animal models to clinical research

The development of the prepulse paradigm using surgical and pharmacological manipulation in rodent models has helped shape clinical understanding for disorders which display sensorimotor gating issues. Davis, Gendelman, Tischler, and Gendelman (1982) identified key forebrain regions which inhibit the startle reflex and this modulatory effect has been shown across all mammals, indicating a translational model for understanding PPI in human research (Swerdlow & Geyer, 1998). PPI impairments have been noted when the CSPT network is damaged, specifically the hippocampus, medial prefrontal cortex and basolateral amygdala (Swerdlow & Geyer, 1998). For example, in rats, damage to the hippocampus with carbachol infusion disrupted PPI, but PPI was not affected when this was infused in the parietal cortex, thus implicating the hippocampus in PPI. Reduced hippocampal volume and abnormal metabolism have been documented in schizophrenia, illustrating the link in abnormalities between animal and clinical research (Harrison, 2004). In addition, damage to the medial prefrontal cortex after 6‐hydroxydopamine lesion in rats causes PPI deficits (Bubser & Koch, 1994), and low levels of dopamine in the prefrontal cortex have also been noted in schizophrenia patients, which would explain the sensorimotor gating issues (Howes & Kapur, 2009).

Research of PPF in animal models is lacking, but some researchers have observed facilitatory effects when rats were injected with ketamine, a NMDA receptor antagonist that can produce a psychosis‐like state in humans (Domino, 1968). Ketamine research supports the notion that clinical psychosis disorders, such as schizophrenia, may be a result of a glutamate deficiency in the hippocampus and prefrontal cortex (Tsai et al., 1995). Therefore, ketamine will reduce PPI and effectively enhance PPF (De Bruin, Ellenbroek, Cools, Coenen, & Van Luijtelaar, 1999; Mansbach & Geyer, 1991). Yet, in human studies, researchers have identified mixed findings in the relationship between PPI and low doses of ketamine, with some studies illustrating an increase in PPI (Abel, Allin, Hemsley, & Geyer, 2003; Duncan et al., 2001), and others a decrease in PPI (van Berckel, Oranje, van Ree, Verbaten, & Kahn, 1998). Using functional neuroimaging techniques combined with pharmacological research could shed further light on differences in neural processing of schizophrenia and the effects of dopamine and glutamate antagonists on the healthy brain, particularly with regard to startle modulation and sensory information processing.

### 5.6 Further avenues to investigate PPI and PPF deficits in clinical disorders

Sensorimotor gating impairments have been linked to psychosis, thus impairments in schizophrenia have been widely documented (e.g., Kumari et al., 2007; Kumari, Gray, Geyer, et al., 2003). Similar impairments have been noted in patients with bipolar disorder who show normal PPI when they are not in the manic phase, which infers a state dependent deficit. Individuals with depression and ADHD, on the other hand, do not show differences in PPI, compared to a healthy control (see Hornix, Havekes, & Kas, 2019). However, in a review by Kohl et al. (2013), several non‐psychotic disorders have documented PPI deficits, including OCD, TS and Huntington's disease. These sensorimotor impairments may be a result of pathophysiology in the neural networks underlying the gating mechanism, thus inhibitory issues may stem from abnormalities in the CSPT circuitry. For example, hyperactivity in the striatum in OCD patients has been linked to PPI deficits (see Stein, 2002), but for autism, anxiety, substance disorders and post‐traumatic stress disorder (PTSD), the PPI literature is unclear (Kohl et al., 2013).

Behavioural research has documented reduced PPF in participants with schizophrenia (Kumari et al., 2007; Storozheva et al., 2012) and in a small number of children with TS (Swerdlow, Karban, et al., 2001), but greater PPF response in women with fibromyalgia, in comparison to female healthy controls (Berryman et al., 2021). However, investigations into PPF differences in clinical groups, compared to healthy controls, are sparse. As neuroimaging research is beginning to investigate whether a shared neural network underlies PPI and PPF, studying PPF in clinical populations that show PPI deficits is essential to establish whether PPF deficits are present, and also to provide further insight into neural dysfunctions which may underly the disorder.

## 6 CONCLUSION

This review has highlighted the existing functional neuroimaging literature on startle modulation using a prepulse paradigm. There was evidence of thalamic, striatal and frontal lobe activation during PPI in healthy groups, and activation deficits at some level in the CSPT circuitry in schizophrenia and TS. The only study to examine both PPI and PPF revealed a shared neural network for PPI and PPF in the frontal regions and cerebellum, with PPF networks also recruiting the superior medial gyrus and cingulate cortex. This paper has highlighted gaps in the limited research on PPF, and investigations of shared PPI‐ and PPF‐related neural regions. Moreover, there is no data on normal sex differences in neural underpinnings of PPI and PPF, nor on neural underpinning of PPI and/or PPF in clinical disorders, other than schizophrenia and TS.

In conclusion, more functional neuroimaging research on PPF is needed to map the neural correlates of startle modulation in healthy and clinical populations. This will allow comparison with consistent and reliable PPI neural networks and illustrate a shared or separate neural network of startle modulation. Ideally, researchers would investigate functional activity of PPI and PPF in discrete experimental blocks during scanning to establish recruitment of shared or separate neural networks with large, mixed sex samples which vary in age for greater validity of findings and to study the effect of sex on neural substrates of PPI and PPF for both healthy populations and clinical populations. Researchers may also wish to study hormonal changes and neural activity during the modulatory effects of startle response.

Further clinical research is needed to study functional neural activity of PPI and PPF in patient populations that demonstrate sensorimotor and attentional deficits, including Huntington's disease, OCD and bipolar disorder, as the current functional neuroimaging literature is primarily focused on schizophrenia. Researchers may wish to compare patient groups with matched samples from different clinical populations to establish similar findings in disorders which display PPI and/or PPF impairments. This has important clinical benefits to understand these disorders further and even highlight PPI and PPF as possible biomarkers of disorders.

## CONFLICT OF INTEREST

The authors declare no conflict of interest.

## FUNDING

Laura F. Naysmith is funded by Lido CTP Unilever, Biotechnology and Biological Sciences Research Council (BBSRC). Steven C. R. Williams would also like to thank the Wellcome Trust and the National Institute for Health Research (NIHR) Maudsley Biomedical Research Centre at South London and Maudsley NHS Foundation Trust and King's College London for their ongoing support of our neuroimaging research.

## Data Availability

Data sharing is not applicable to this article as no new data were created or analyzed in this study.
